# Supplementary material for: Self-assembly of acetate adsorbates drives atomic rearrangement on the Au(110) surface
Source: Nat Commun. 2016 Oct 12;7:13139. doi: 10.1038/ncomms13139 (PMC5064018; doi:10.1038/ncomms13139)
Supplement: Supplementary Information — Supplementary Figures 1-7, Supplementary Tables 1-6, Supplementary Discussion and Supplementary References. [file ncomms13139-s1.pdf]

Supplementary Figures:

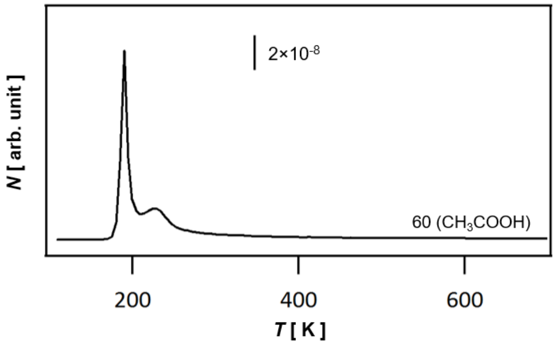

Supplementary Figure 1: Temperature programmed desorption of acetic acid (*m*/*z*=60).

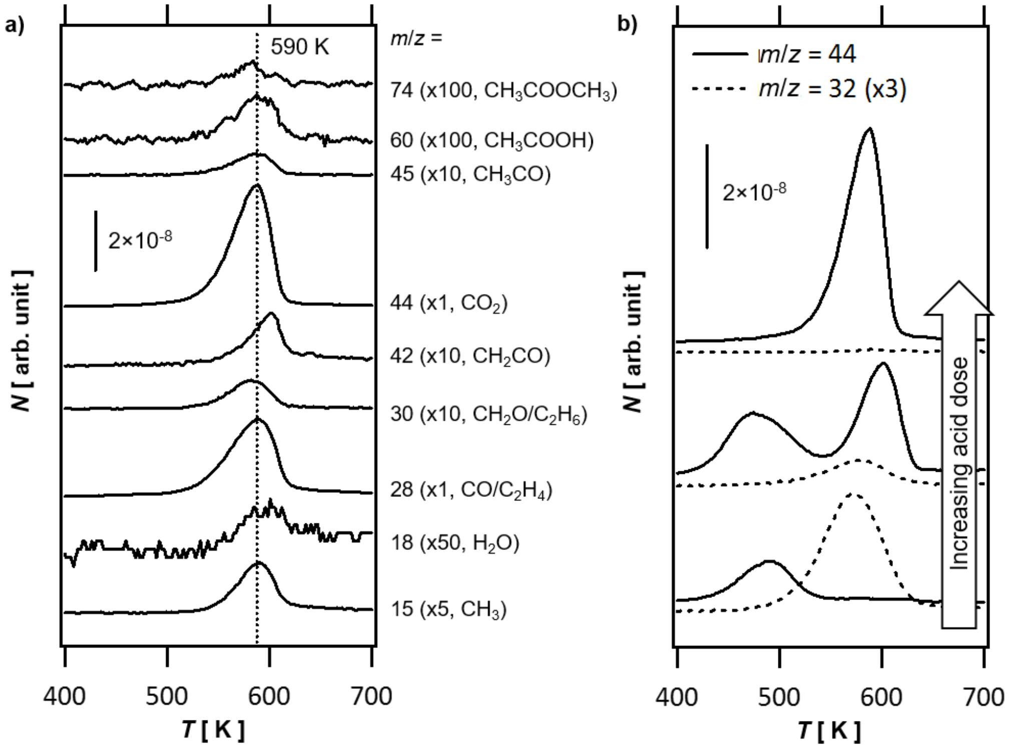

Supplementary Figure 2: Temperature programmed reaction of acetate on Au(110) precovered with oxygen (Initial O_{ads} = 0.14 ML). (a) Decomposition products in excess acid condition i.e. no coadsorbed O_{ads}. (b) Evolution of the decomposition temperature with increasing acid/oxygen ratio monitored via the main product CO (*m*/*z*=44) and remaining unreacted O (*m*/*z*=32). Temperature ramp rate = 5 K/s.

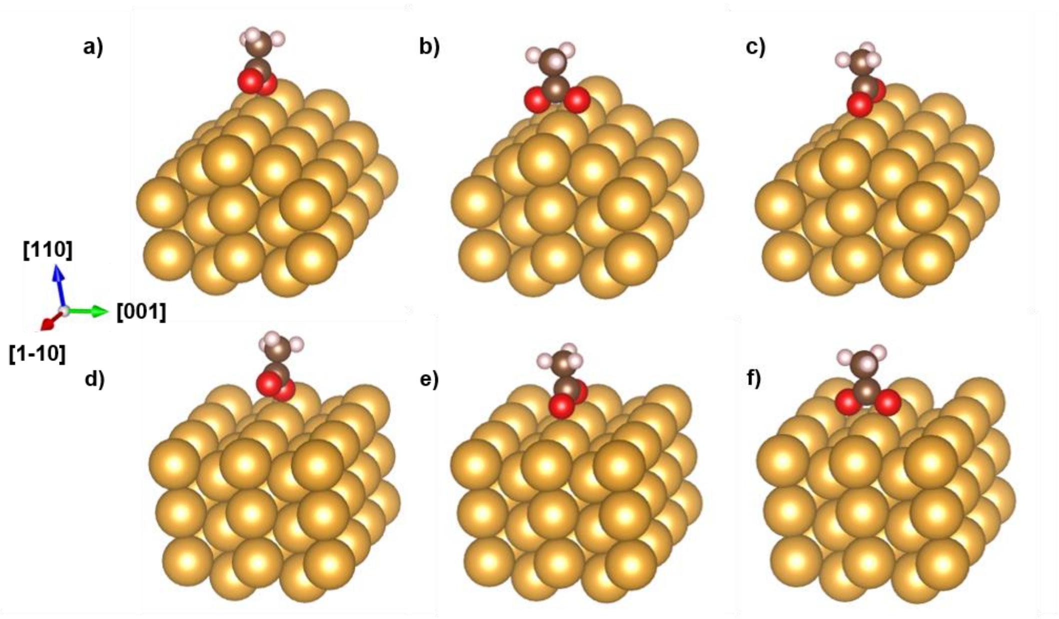

Supplementary Figure 3: Explored acetate adsorption configurations. Bridge monodentate, chelating and bidentate top on Au-(1×2) (a,b,c) and on Au-(1×1) (d, e, f). All final atomic positions are given in Supplementary Data 1.

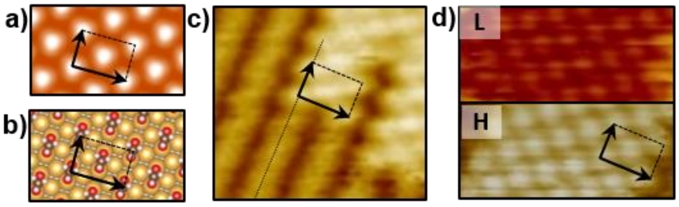

Supplementary Figure 4: STM images versus DFT-simulated images. (a) Simulated constant-height STM image (2.5 Å above adsorbates, +1.5 V). (b) Corresponding relaxed c(2×2) bidentate acetate geometry. (c) and (d) STM images showing molecular resolution on the acetate layer at low-coverage (0.05 ML) and saturation coverage (0.25 ML). Arrows indicate the c(2×2) unit cell parameters (5.5×8.2 $Å^2$).

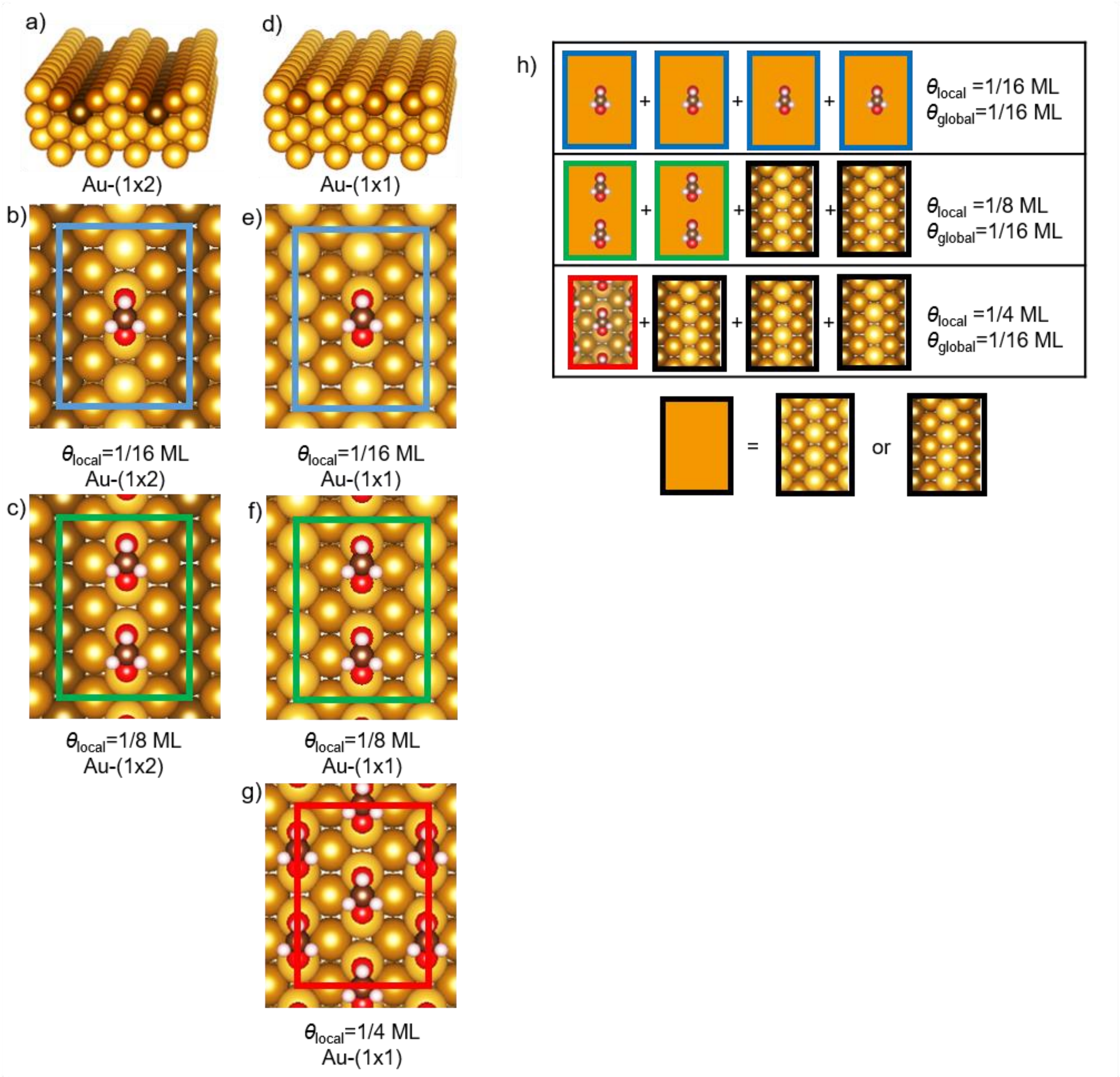

Supplementary Figure 5: Supercells and supercell combinations considered in the calculation of the interface energy per acetate. (a-g) Representation of the supercells, for all 5 acetate coverage-Au interface structure combinations. (h) Schematic representation of the combinations of supercell total energy used in the calculation of the interface energy per acetate in Eq. 2. Supercells (b-c, e-g) are combined with clean Au-(1×2) supercells which allows us to investigate various local coverages (θ_{local}) at constant global coverage of $\theta_{global=}$ 1/16 ML. All final atomic positions are given in Supplementary Data 1.

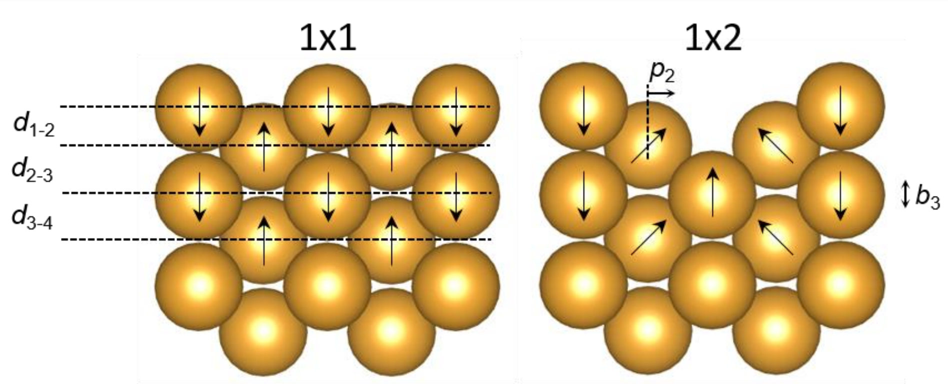

Supplementary Figure 6: Schematic of the relaxation of pure Au-(1×1) and Au-(1×2).

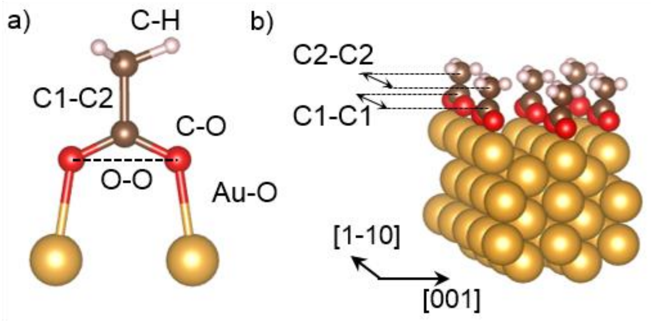

Supplementary Figure 7: Relevant intramolecular and intermolecular distances. Schematic of the acetate bond lengths and distances within the acetate molecule (a) and between acetate nearest neighbors along the row direction (b).

Supplementary Tables

Adsorbate $B_{(ads)}$	Reaction Energy (-Δ*E*) on Au-(1×2) (eV)	
	**PBE[(a)]**	**PBE+vdW[(b)]**
**CH_3CH_2O (ethoxy)**	0.21	0.55
**CH_3COO (acetate)**	0.84	1.00

Supplementary Table 1: Reaction energy (cf. Supplementary Equation 1) of adsorption of ethoxy and acetate using (a) PBE relaxed and (b) DFT-TS (vdW included) relaxed geometries.

	**Monodentate Bridge**		**Chelating Top**		**Bidentate Dual top**	
**Au-(1×2)**	**vdW**	**PBE**	**vdW**	**PBE**	**vdW**	**PBE**
**Au-(1×1)**	relaxed into bidentate dual top		-2.00	-1.77	-2.62	-2.42
			-2.03	-1.75	-2.59	-2.36

Supplementary Table 2: Adsorption energy of acetate (eV) calculated using Eq. 3 for various configurations on both Au-(1×2) and Au-(1×1) interfaces (Supplementary Figure 2). The columns labeled vdW refer to the energies calculates when van der Waals interactions are included in the PBE calculation; PBE refers to pure PBE calculations.

	Au-(1×2)				Au-(1×1)			
	vdW		PBE		PBE		PBE	
**Δ*d*$_{1-2}$** (Å, %)	-0.32	-22.0	-0.33	-22.0	-0.16	-11.0	-0.21	-14.2
**Δ*d*$_{2-3}$** (Å, %)	0.08	5.3	0.04	5.3	0.13	9.0	0.14	9.6
**Δ*d*$_{3-4}$** (Å, %)	-0.01	-1.0	-0.01	-1.0	-0.06	-3.8	-0.06	-4.0
***b*$_3$** (Å)	0.27		0.31					
***p*$_2$** (Å)	0.07		0.05					

Supplementary Table 3: Relaxation in Å and in % compared to the ideal Au-(1×2) and Au-(1×1) structures, distances are labelled on Supplementary Figure 6.

	θ_{local}=1/16 ML							
	Au-(1×2)				Au-(1×1)			
	vdW		PBE		vdW		PBE	
**Δ*d*$_{1-2}$** (Å, %)	0.08	6.9	0.11	9.9	0.01	0.62	0.01	1.1
**Δ*d*$_{2-3}$** (Å, %)	0.01	0.68	0.01	0.33	-0.09	-5.7	-0.12	-7.4
**Δ*d*$_{3-4}$** (Å, %)	-0.04	-2.9	-0.04	-2.6	0.08	5.7	0.10	7.4
***b*$_3$** (Å)	-0.10		-0.13					

Supplementary Table 4: Relaxation of the Au atoms upon adsorption of isolated (θ_{local}=1/16 ML) acetate (in Å and in %) compared to the relaxed pure Au-(1×2) and Au-(1×1) surfaces, distances as labelled on Supplementary Figure 6. For isolated acetate, we focus on atoms that relax by more than 0.05 Å within a specific layer. If relaxation falls under this threshold, the average position over the cell is considered.

	θ_{local} =1/8 ML								θ_{local} =1/4 ML			
	Au-(1×2)				Au-(1×1)				Au-(1×1)			
	vdW		PBE		vdW		PBE		vdW		PBE	
**Δd1-2** (Å, %)	0.08	7.1	0.11	9.6	0.01	0.62	-0.01	-0.79	0.02	1.8	0.06	4.4
**Δd2-3** (Å, %)	-0.08	-5.2	-0.09	-6.1	-0.06	-3.8	-0.08	-4.7	-0.1	-6.3	-0.12	-7.6
**Δd3-4** (Å, %)	0.04	2.9	0.05	3.7	0.03	2.0	0.03	2.3	0.06	4.2	0.07	5.1
**Δb3** (Å)	-0.11		-0.13									

Supplementary Table 5: Relaxation of the Au atoms upon adsorption of acetate in the θ_{local} =1/8 ML and θ_{local} =1/4 ML geometries (in Å and in %) compared to the relaxed pure Au-(1×2) and Au-(1×1) surfaces, distances as labelled on Supplementary Figure 6.

	θ_{local}=1/16 ML				θ_{local}=1/8 ML				θ_{local}=1/4 ML		Isolated Gas phase	
	Au-(1×2)		Au-(1×1)		Au-(1×2)		Au-(1×1)		Au-(1×1)			
	vdW	PBE	vdW	PBE	vdW	PBE	vdW	PBE	vdW	PBE	vdW	PBE
**Au-O**	2.207	2.212	2.198	2.206	2.198	2.203	2.192	2.199	2.189	2.197	N/A	N/A
**O-C**	1.276	1.276	1.276	1.276	1.276	1.276	1.275	1.276	1.275	1.275	1.239	1.239
**C-C**	1.516	1.517	1.515	1.517	1.516	1.517	1.516	1.517	1.515	1.517	1.65	1.649
**O-C-O**	128	128	128	128	128	128	128	128	128	128	141	141
**C-H**	1.098	1.098	1.097	1.098	1.098	1.098	1.098	1.098	1.098	1.098	1.094	1.094
**C1-C1**	N/A	N/A	N/A	N/A	5.817	5.877	5.818	5.879	5.817	5.877	N/A	N/A
**C2-C2**	N/A	N/A	N/A	N/A	5.817	5.877	5.819	5.880	5.817	5.877	N/A	N/A

Supplementary Table 6: Bond distances (Å) and O-C-O angle (˚) labelled in Supplementary Figure 7, for all bidentate top adsorption configurations considered (see Supplementary Figure 1), compared to gas phase relaxation of neutral acetate.

Supplementary Discussion

The poisoning potential of acetate: the case of ethanol self-coupling

In order to evaluate the poisoning potential of acetate to ethanol self-coupling on Au(110), the stability of acetate and ethoxy are compared based on the deprotonation reaction on O-precovered Au(110):

$$2BH_{(gas)} + O_{(ads)} \rightarrow 2B_{(ads)} + H_2O_{(gas)}, \qquad (1)$$

where BH is the acetic acid or ethanol and $B_{(ads)}$ is the resulting adsorbate acetate or ethoxy. In this calculation, non-interacting adsorbates are considered. Supplementary Table 1 shows that acetate is more stable than ethoxy on the Au(110), confirming its site-blocking potential to ethoxy adsorption, which is the necessary initial step in ethanol self-coupling.

Thermal decomposition of acetate on Au(110)

As a preliminary to the study of acetate decomposition on Au(110), we confirm that acetic acid does not react with the clean Au(110) surface and that the acid desorbs from the surface well below room temperature. Supplementary Figure 1 shows the temperature programmed desorption experiment after multilayer condensation of acetic acid at low temperature. Two desorption peaks are detected at 190 K and 226 K. Hence, acetic acid is not present on the surface in the room temperature STM experiments.

Consistently with the calculated high adsorption energy, acetate is thermally stable on Au(110) to above 500 K when there is no oxygen co-adsorbed on the surface (Supplementary Figure 2a). Under this condition, the main products of acetate decomposition are gaseous CO_2 and CH_3, peaking at 590 K. In the presence of remaining coadsorbed oxygen (Supplementary Figure

2b), a second decomposition channel appears, peaking at 490 K. It is however possible to still observe the high temperature decomposition in excess oxygen condition. A detailed study of the effect of adsorbed oxygen on the reactivity and structure will be described elsewhere.

Acetate adsorption configuration

Among all adsorption structures considered (Supplementary Figure 3), the preferred binding configuration for isolated acetate (θ_{local}=1/16 ML) is the bidentate configuration in which the O-C-O plane is perpendicular to the surface. This result is obtained from the acetate adsorption energies (Eq. 3) in Supplementary Table 2.

At this low coverage limit, the monodentate bridge structure relaxes into the preferred bidentate dual top; the chelating top geometry is less stable by more than 0.6 eV (58 kJ/mol). The relative stabilities of these various geometries in the low coverage limit is essentially the same whether van der Waals (vdW) interactions are included or not; however, the absolute binding energies are increased ~0.2 eV by these weak interactions.

Finally, bidentate acetate is preferred on both the Au-(1×1) and Au-(1×2) surfaces. Note that the adsorption energy of acetate on each of those interfaces cannot be compared directly. The interface energy defined in Eq. 2 has to be used in order to correctly take into account the energy cost of the Au-(1×2) to Au-(1×1) transformation.

Simulation of STM images

The simulation of STM images (Supplementary Figure 4a) using the bidentate top c(2×2) acetate molecular ordering on Au-(1×1) (Supplementary Figure 4b) supports the interpretation of the experimental protrusions (Supplementary Figure 4c,d) arising from the acetate methyl group.

Distortions of the Au surface upon acetate adsorption

Pure Au relaxation

The relaxation of pure Au(110)-(1×2) and Au(110)-(1×1) surfaces (Supplementary Figure 6 and Supplementary Table 3) is consistent with previous studies, yielding a contraction of the top three atomic layers.[1,2]

Au further relaxation upon adsorption of acetate

Upon acetate adsorption, the general trend in the relaxation of the Au surface is to revert the contraction of atomic layers discussed above. Lateral displacements are lower than ~0.05 Å. The detailed analysis of the evolution of the interlayer spacing is given in Supplementary Tables 4,5.

Acetate bond analysis for all bidentate configurations

Surface adsorption induces changes in acetate C-O (+3%) and C-C (-8%) bond length and O-O distance (-2%) compared to gas phase calculations. Those values are independent from the Au surface reconstruction considered and the acetate coverage. The main effect of vdW interactions is O-Au bond contraction, by less than 1%, consistently with an increased Au-molecule interaction. The bond distances, labelled in Supplementary Figure 7, are given in Supplementary Table 6.

Supplementary References:

1.Landmann, M., Rauls, E. & Schmidt, W. G. First-principles calculations of clean Au (110) surfaces and chemisorption of atomic oxygen. *Phys Rev B* **79,** 045412-045421 (2009).

2.Dos Reis, D. D., Negreiros, F. R., De Carvalho, V. E. & Soares, E. A. Geometry of the Au(110)-(1×2) missing-row clean surface: A New LEED and DFT study. *Surf Sci* **604,** 568-573 (2010).
