# Supplementary material for: Self-assembly of acetate adsorbates drives atomic rearrangement on the Au(110) surface
Source: Nat Commun. 2016 Oct 12;7:13139. doi: 10.1038/ncomms13139 (PMC5064018; doi:10.1038/ncomms13139)
Supplement: Supplementary Data 1 — Atomic coordinates from DFT calculations [file ncomms13139-s2.docx]

**Supplementary Data 1:**

**Atomic coordinates from DFT calculations**

List of structures:

1 PBE relaxed structures:

1.1 Chelating 1×2

1.2 Chelating 1×1

1.3 Bidentate top 1×2

1.4 Bidentate top 1×1

1.5 Bidentate (2×2) 1×2

1.6 Bidentate (2×2) 1×1

1.7 Bidentate c(2×2) 1×1

2 PBE-TS relaxed structures

2.1 Chelating 1×2

2.2 Chelating 1×1

2.3 Bidentate top 1×2

2.4 Bidentate top 1×1

2.5 Bidentate (2×2) 1×2

2.6 Bidentate (2×2) 1×1

2.7 Bidentate c(2×2) 1×1

# PBE relaxed structures:

## Chelating 1×2

4.15597938929980

2.8284271247461801 0.0000000000000000 0.0000000000000000

0.0000000000000000 2.0000000000000000 0.0000000000000000

0.0000000000000000 0.0000000000000000 5.3033008588991004

O C H Au

2 2 3 44

Selective dynamics

Direct

0.0000000000000000 0.1321728758702179 0.4243127366881367 T T T

0.0000000000000000 0.8633770246564083 0.4242091344467767 T T T

0.0000000000000000 0.9975298352370885 0.4523936162400304 T T T

0.0000000000000000 0.9986736092157571 0.5209314155419023 T T T

0.0000000000000000 0.8758554620869603 0.5390477476182554 T T T

0.9247215758677712 0.0640133695589711 0.5373501972473562 T T T

0.0752784241322360 0.0640133695589711 0.5373501972473562 T T T

0.0000000000000000 0.0004459853870529 0.3289803248309582 T T T

0.2540324444842454 0.0002871649835114 0.3208989424651065 T T T

0.1248595255991276 0.2501680935195125 0.2675013353515183 T T T

0.3757424004997389 0.2584604490221309 0.2697935790552748 T T T

0.1248464865362209 0.7504405837061441 0.2674611720210507 T T T

0.3757274129689279 0.7420080044201853 0.2697972095388153 T T T

0.0000000000000000 0.0001234621482951 0.1990703356964474 T T T

0.2502954134524273 0.0001373343277749 0.1941515378339034 T T T

0.0000000000000000 0.5001899002938686 0.2083374619712401 T T T

0.2484559847135941 0.5001945206553273 0.2087339995348714 T T T

0.1240139661739192 0.2569008785678619 0.1339520114346939 T T T

0.3743597728916086 0.2589426985029846 0.1347753280825899 T T T

0.1240272377192326 0.7432018960402758 0.1339316468690939 T T T

0.3743694482284710 0.7412086240063616 0.1347689051887855 T T T

0.0000000000000000 0.0000000000000000 0.0666666666666700 F F F

0.2500000000000000 0.0000000000000000 0.0666666666666700 F F F

0.0000000000000000 0.5000000000000000 0.0666666666666700 F F F

0.2500000000000000 0.5000000000000000 0.0666666666666700 F F F

0.1250000000000000 0.2500000000000000 0.0000000000000000 F F F

0.3750000000000000 0.2500000000000000 0.0000000000000000 F F F

0.1250000000000000 0.7500000000000000 0.0000000000000000 F F F

0.3750000000000000 0.7500000000000000 0.0000000000000000 F F F

0.5000000000000000 0.0002397486816434 0.3212049399840708 T T T

0.7459675555157617 0.0002871649835114 0.3208989424651065 T T T

0.6242575995002611 0.2584604490221309 0.2697935790552748 T T T

0.8751404744008724 0.2501680935195125 0.2675013353515183 T T T

0.6242725870310721 0.7420080044201853 0.2697972095388153 T T T

0.8751535134637791 0.7504405837061441 0.2674611720210507 T T T

0.5000000000000000 0.0001143177853180 0.1940251118488519 T T T

0.7497045865475727 0.0001373343277749 0.1941515378339034 T T T

0.5000000000000000 0.5002058749261664 0.2071987419698900 T T T

0.7515440152864059 0.5001945206553273 0.2087339995348714 T T T

0.6256402271083914 0.2589426985029846 0.1347753280825899 T T T

0.8759860338260808 0.2569008785678619 0.1339520114346939 T T T

0.6256305517715290 0.7412086240063616 0.1347689051887855 T T T

0.8759727622807674 0.7432018960402758 0.1339316468690939 T T T

0.5000000000000000 0.0000000000000000 0.0666666666666700 F F F

0.7500000000000000 0.0000000000000000 0.0666666666666700 F F F

0.5000000000000000 0.5000000000000000 0.0666666666666700 F F F

0.7500000000000000 0.5000000000000000 0.0666666666666700 F F F

0.6250000000000000 0.2500000000000000 0.0000000000000000 F F F

0.8750000000000000 0.2500000000000000 0.0000000000000000 F F F

0.6250000000000000 0.7500000000000000 0.0000000000000000 F F F

0.8750000000000000 0.7500000000000000 0.0000000000000000 F F F

## Chelating 1×1

4.15597938929980

2.8284271247461801 0.0000000000000000 0.0000000000000000

0.0000000000000000 2.0000000000000000 0.0000000000000000

0.0000000000000000 0.0000000000000000 5.3033008588991004

O C H Au

2 2 3 48

Selective dynamics

Direct

-0.0000000400836725 0.1279000909505653 0.4296826937794017 T T T

0.0000000135274448 0.8590418930846558 0.4308370731886143 T T T

-0.0000000430728773 0.9937424773091065 0.4583524656682890 T T T

-0.0000000835619449 0.9980529018653040 0.5269207832079122 T T T

-0.0000001125819335 0.8761126555140574 0.5457715498800367 T T T

0.9247215293419222 0.0641178519038955 0.5428971961578211 T T T

0.0752783039289468 0.0641178337541368 0.5428972099762536 T T T

0.0000004083158646 0.0005643471817897 0.3345208300036669 T T T

0.2552075573448989 0.0004918578963719 0.3283000821652047 T T T

0.1252858104661814 0.2478045466820298 0.2713662061646083 T T T

0.3758896894818415 0.2520056577485180 0.2715776854313571 T T T

0.1252329262486654 0.7534546388254748 0.2713392492462251 T T T

0.3759076558638523 0.7488397880193031 0.2715368990039976 T T T

0.0000003652894919 0.0002324471360759 0.2037019504262508 T T T

0.2500731334885012 0.0002790218052860 0.1989745614768901 T T T

-0.0000000393028620 0.5001805495391024 0.1986567214708716 T T T

0.2492126091065703 0.5002727153908703 0.1984748305638834 T T T

0.1242739863469039 0.2484995563968783 0.1346574717390077 T T T

0.3749261078195977 0.2502241088725411 0.1350618718659516 T T T

0.1242710974205179 0.7516412972411465 0.1345831637790647 T T T

0.3749441343113243 0.7501182735476158 0.1350553234818135 T T T

0.0000000000000000 0.0000000000000000 0.0666666666666700 F F F

0.2500000000000000 0.0000000000000000 0.0666666666666700 F F F

0.0000000000000000 0.5000000000000000 0.0666666666666700 F F F

0.2500000000000000 0.5000000000000000 0.0666666666666700 F F F

0.1250000000000000 0.2500000000000000 0.0000000000000000 F F F

0.3750000000000000 0.2500000000000000 0.0000000000000000 F F F

0.1250000000000000 0.7500000000000000 0.0000000000000000 F F F

0.3750000000000000 0.7500000000000000 0.0000000000000000 F F F

0.0000002494753059 0.5007300267332893 0.3276206804504829 T T T

0.2489589971824142 0.5006307578633530 0.3279125726912946 T T T

0.4999997654474618 0.5004136461797728 0.3298691532000286 T T T

0.7510410700908856 0.5006303937816775 0.3279126466659402 T T T

0.5000004771001624 0.0004485198874050 0.3297939021497905 T T T

0.7447933674320720 0.0004887320256257 0.3283000316027459 T T T

0.6241098421081214 0.2520051606136504 0.2715781040027502 T T T

0.8747145136876417 0.2478044227372272 0.2713662910521726 T T T

0.6240913937357145 0.7488405956298696 0.2715369408880343 T T T

0.8747681614468537 0.7534541507109109 0.2713392240635666 T T T

0.4999998355086125 0.0003156311543737 0.1990307248227316 T T T

0.7499266679522466 0.0002790450624348 0.1989744449769239 T T T

0.4999996018771734 0.5002600607979998 0.1988753539072254 T T T

0.7507873932247554 0.5002720032893326 0.1984746566102588 T T T

0.6250731624977899 0.2502239541007730 0.1350619439358614 T T T

0.8757264082781140 0.2484993785121395 0.1346574881247529 T T T

0.6250555822655731 0.7501182519258629 0.1350550565319370 T T T

0.8757298078069198 0.7516414331878224 0.1345831740320333 T T T

0.5000000000000000 0.0000000000000000 0.0666666666666700 F F F

0.7500000000000000 0.0000000000000000 0.0666666666666700 F F F

0.5000000000000000 0.5000000000000000 0.0666666666666700 F F F

0.7500000000000000 0.5000000000000000 0.0666666666666700 F F F

0.6250000000000000 0.2500000000000000 0.0000000000000000 F F F

0.8750000000000000 0.2500000000000000 0.0000000000000000 F F F

0.6250000000000000 0.7500000000000000 0.0000000000000000 F F F

0.8750000000000000 0.7500000000000000 0.0000000000000000 F F F

## Bidentate top 1×2

4.15597938929980

2.8284271247461801 0.0000000000000000 0.0000000000000000

0.0000000000000000 2.0000000000000000 0.0000000000000000

0.0000000000000000 0.0000000000000000 5.3033008588991004

O C H Au

2 2 3 44

Selective dynamics

Direct

0.2217959679804415 -0.0000000174600344 0.4234039717295308 T T T

0.0266315998372740 0.0000000297714035 0.4232717315089298 T T T

0.1241333251018581 -0.0000000081853021 0.4487223597592989 T T T

0.1249115882428394 0.0000000126106406 0.5175487813121306 T T T

0.0381960218206092 -0.0000000489401470 0.5356790940603938 T T T

0.1712465801185004 0.8935265375160822 0.5337809993405528 T T T

0.1712465603921841 0.1064733417385422 0.5337810052686489 T T T

0.9992859033699525 0.0000000058349433 0.3239861523604868 T T T

0.2503593743483120 0.0000000257069674 0.3242068966293936 T T T

0.1248918078576366 0.2552974653260016 0.2666223939347038 T T T

0.3755269160108595 0.2540690212423059 0.2677670614733095 T T T

0.1248918237679893 0.7447025650906610 0.2666223855149671 T T T

0.3755269121466808 0.7459309340354879 0.2677670838930371 T T T

0.9989779456916873 0.0000000146887657 0.1983893892741485 T T T

0.2508332883140260 -0.0000000111652485 0.1984930879118927 T T T

0.9994555249439603 0.5000000112622509 0.2068446479396795 T T T

0.2503900865517494 0.4999999971863019 0.2068523079823667 T T T

0.1249789704472221 0.2542803683078266 0.1337175801747185 T T T

0.3739812173972833 0.2565118256150445 0.1337233133729207 T T T

0.1249789614733128 0.7457196326695983 0.1337175937614736 T T T

0.3739812073851770 0.7434881830889639 0.1337232931847106 T T T

0.0000000000000000 0.0000000000000000 0.0666666666666700 F F F

0.2500000000000000 0.0000000000000000 0.0666666666666700 F F F

0.0000000000000000 0.5000000000000000 0.0666666666666700 F F F

0.2500000000000000 0.5000000000000000 0.0666666666666700 F F F

0.1250000000000000 0.2500000000000000 0.0000000000000000 F F F

0.3750000000000000 0.2500000000000000 0.0000000000000000 F F F

0.1250000000000000 0.7500000000000000 0.0000000000000000 F F F

0.3750000000000000 0.7500000000000000 0.0000000000000000 F F F

0.5021197496692427 -0.0000000253913882 0.3200313004241829 T T T

0.7475419427728203 0.0000000062576535 0.3199755146240565 T T T

0.6248624414521065 0.2600496790505173 0.2698071771996167 T T T

0.8741473196703664 0.2542123167329713 0.2677184371927759 T T T

0.6248624491148944 0.7399503030963145 0.2698071791219845 T T T

0.8741473244719143 0.7457877061954560 0.2677184326368640 T T T

0.5004406250615301 -0.0000000069657574 0.1936627882434187 T T T

0.7494273324857410 -0.0000000336130771 0.1936395889990624 T T T

0.4995314663336259 0.5000000092831185 0.2072084849361821 T T T

0.7501579093496473 0.5000000119412984 0.2071416577717319 T T T

0.6249259461287210 0.2585862983353145 0.1344280521580754 T T T

0.8759277514822270 0.2565524335057440 0.1337033299947187 T T T

0.6249259253623926 0.7414137097095452 0.1344280299606444 T T T

0.8759277450854700 0.7434475809554624 0.1337032985552868 T T T

0.5000000000000000 0.0000000000000000 0.0666666666666700 F F F

0.7500000000000000 0.0000000000000000 0.0666666666666700 F F F

0.5000000000000000 0.5000000000000000 0.0666666666666700 F F F

0.7500000000000000 0.5000000000000000 0.0666666666666700 F F F

0.6250000000000000 0.2500000000000000 0.0000000000000000 F F F

0.8750000000000000 0.2500000000000000 0.0000000000000000 F F F

0.6250000000000000 0.7500000000000000 0.0000000000000000 F F F

0.8750000000000000 0.7500000000000000 0.0000000000000000 F F F

## Bidentate top 1×1

4.15597938929980

2.8284271247461801 0.0000000000000000 0.0000000000000000

0.0000000000000000 2.0000000000000000 0.0000000000000000

0.0000000000000000 0.0000000000000000 5.3033008588991004

O C H Au

2 2 3 48

Selective dynamics

Direct

0.2217954472991087 0.0000000666690498 0.4298813332937485 T T T

0.0266694616626188 -0.0000001087521891 0.4298090779265016 T T T

0.1241850626497528 0.0000000079922760 0.4552165649904790 T T T

0.1250450021860572 -0.0000001053092076 0.5240512859818768 T T T

0.0383208219570468 0.0000000007375065 0.5421868779233840 T T T

0.1714150406388308 0.8935160240725324 0.5402536815776378 T T T

0.1714150555876081 0.1064841223455943 0.5402536922127934 T T T

0.9987337863344294 -0.0000003160039639 0.3308558409267943 T T T

0.2507798973874200 -0.0000001320844211 0.3310146463777007 T T T

0.4988929827055512 0.5000000608589182 0.3285758869505268 T T T

0.2501630661809127 0.4999999054120201 0.3274779548597440 T T T

0.1248748360108343 0.2515886865529963 0.2703190791722632 T T T

0.3758073857561827 0.2490620157475937 0.2713110049164260 T T T

0.1248760791297608 0.7484100087137677 0.2703181745385638 T T T

0.3758074915244358 0.7509380782692927 0.2713109260635687 T T T

0.5002666537436395 0.0000000496353327 0.1985672348241712 T T T

0.2507784551892158 0.0000001720608105 0.2034116275943703 T T T

0.4996088572222319 0.5000000565700603 0.1982769737008693 T T T

0.2502577061889771 0.4999998493948850 0.1977390138623079 T T T

0.1249755685332573 0.2462110764085522 0.1347296912821468 T T T

0.3742725963853968 0.2484799244129850 0.1345190886929015 T T T

0.1249755634996398 0.7537890693637076 0.1347295602121788 T T T

0.3742726544740166 0.7515201908387970 0.1345190133887134 T T T

0.0000000000000000 0.0000000000000000 0.0666666666666700 F F F

0.2500000000000000 0.0000000000000000 0.0666666666666700 F F F

0.0000000000000000 0.5000000000000000 0.0666666666666700 F F F

0.2500000000000000 0.5000000000000000 0.0666666666666700 F F F

0.1250000000000000 0.2500000000000000 0.0000000000000000 F F F

0.3750000000000000 0.2500000000000000 0.0000000000000000 F F F

0.1250000000000000 0.7500000000000000 0.0000000000000000 F F F

0.3750000000000000 0.7500000000000000 0.0000000000000000 F F F

0.5029389469259079 0.0000000158927767 0.3282948020811585 T T T

0.7466866871571808 0.0000000537047592 0.3282060666113367 T T T

0.9995324065110557 0.5000001208853155 0.3274595853071362 T T T

0.7507206753400352 0.5000000094347645 0.3285798211747577 T T T

0.6248042779089354 0.2524374875712773 0.2711569217883874 T T T

0.8738524009130095 0.2492076004763627 0.2712533411671071 T T T

0.6248042984113829 0.7475625820599459 0.2711569971847845 T T T

0.8738525230450420 0.7507924637087136 0.2712533270291970 T T T

0.9989898422304275 -0.0000000006860692 0.2033446267239123 T T T

0.7494909283558653 0.0000000175095760 0.1985456948876746 T T T

0.9996318672347158 0.4999999521676759 0.1977157436954550 T T T

0.7502183135550985 0.5000000490940230 0.1982669415258201 T T T

0.6249490012447506 0.2503440697284500 0.1347988726976270 T T T

0.8756390272299261 0.2484729190495791 0.1344979304971267 T T T

0.6249490057224562 0.7496559848131582 0.1347988921561943 T T T

0.8756389937819846 0.7515271682027829 0.1344978735539774 T T T

0.5000000000000000 0.0000000000000000 0.0666666666666700 F F F

0.7500000000000000 0.0000000000000000 0.0666666666666700 F F F

0.5000000000000000 0.5000000000000000 0.0666666666666700 F F F

0.7500000000000000 0.5000000000000000 0.0666666666666700 F F F

0.6250000000000000 0.2500000000000000 0.0000000000000000 F F F

0.8750000000000000 0.2500000000000000 0.0000000000000000 F F F

0.6250000000000000 0.7500000000000000 0.0000000000000000 F F F

0.8750000000000000 0.7500000000000000 0.0000000000000000 F F F

## Bidentate (2×2) 1×2

4.15597938929980

2.8284271247461801 0.0000000000000000 0.0000000000000000

0.0000000000000000 2.0000000000000000 0.0000000000000000

0.0000000000000000 0.0000000000000000 5.3033008588991004

O C H Au O C H Au

2 2 3 22 2 2 3 22

Selective dynamics

Direct

0.2166603500003172 -0.0000023217892335 0.4221885183060348 T T T

0.5215363671823210 0.0000027850887163 0.4216100054309934 T T T

0.1188449774259711 0.0000027458936023 0.4472694797528760 T T T

0.1187444460131678 -0.0000023595178826 0.5160816461150337 T T T

0.5316474241103734 0.0000041850239950 0.5337000632054189 T T T

0.1648316264527198 0.8934847000057092 0.5324563419522089 T T T

0.1648313970299932 0.1065140443615217 0.5324562545961461 T T T

0.4972560687939204 0.0000024991761677 0.3226721892055767 T T T

0.2447033837837025 0.9999984077320345 0.3232038499944409 T T T

0.1216185306183162 0.2581666123017947 0.2665717018031843 T T T

0.3714264335203410 0.2531571970353031 0.2661240718657697 T T T

0.1216180532061423 0.7418322496841739 0.2665727716374752 T T T

0.3714268939414982 0.7468479068129538 0.2661224893441530 T T T

0.4973612623434811 0.0000019754214890 0.1977631274394316 T T T

0.2473771072497069 0.0000033950806739 0.1979888025537791 T T T

0.4964336662808193 0.5000008946694428 0.2058677783456422 T T T

0.2477497676244406 0.5000006203570209 0.2057973181971113 T T T

0.1237943238066069 0.2546001626509159 0.1334906154652458 T T T

0.3737999650588711 0.2546717077799423 0.1329494215170538 T T T

0.1237956110488750 0.7453979590925129 0.1334901886042089 T T T

0.3737987804344709 0.7453318288451573 0.1329499702918774 T T T

0.0000000000000000 0.0000000000000000 0.0666666666666700 F F F

0.2500000000000000 0.0000000000000000 0.0666666666666700 F F F

0.0000000000000000 0.5000000000000000 0.0666666666666700 F F F

0.2500000000000000 0.5000000000000000 0.0666666666666700 F F F

0.1250000000000000 0.2500000000000000 0.0000000000000000 F F F

0.3750000000000000 0.2500000000000000 0.0000000000000000 F F F

0.1250000000000000 0.7500000000000000 0.0000000000000000 F F F

0.3750000000000000 0.7500000000000000 0.0000000000000000 F F F

0.7166576481295907 0.0000056743003074 0.4221817057850017 T T T

0.0215484140513223 -0.0000004733480794 0.4216203795759102 T T T

0.6188360454913581 -0.0000031564078530 0.4472674125642775 T T T

0.6187271376546234 0.0000069215359704 0.5160752882863595 T T T

0.0316597916279617 0.9999990651955385 0.5337081074186779 T T T

0.6648161174041406 0.8934879355935502 0.5324539497554543 T T T

0.6648160170838038 0.1065208054882915 0.5324540200277148 T T T

0.9972576725642762 -0.0000008815615508 0.3226740199396559 T T T

0.7447079681710825 0.0000017049891070 0.3231960338275279 T T T

0.6216183523651310 0.2581741119434733 0.2665802851842027 T T T

0.8714263220005164 0.2531617792396527 0.2661315682085207 T T T

0.6216194687564359 0.7418261036498939 0.2665806229616357 T T T

0.8714232259714828 0.7468243581314619 0.2661377134267821 T T T

0.9973444946392290 0.9999924391135969 0.1977689472817593 T T T

0.7473908774799922 0.9999964134710810 0.1979861723249363 T T T

0.9964248895386426 0.4999999923564729 0.2058681538440041 T T T

0.7477484721782219 0.4999976875267074 0.2058026917608466 T T T

0.6238043978359540 0.2546082622785881 0.1334952257868293 T T T

0.8737794499784239 0.2546711591569343 0.1329524646824167 T T T

0.6238044944961767 0.7453900993578901 0.1334949785746041 T T T

0.8737791099738943 0.7453276903434227 0.1329547318365532 T T T

0.5000000000000000 0.0000000000000000 0.0666666666666700 F F F

0.7500000000000000 0.0000000000000000 0.0666666666666700 F F F

0.5000000000000000 0.5000000000000000 0.0666666666666700 F F F

0.7500000000000000 0.5000000000000000 0.0666666666666700 F F F

0.6250000000000000 0.2500000000000000 0.0000000000000000 F F F

0.8750000000000000 0.2500000000000000 0.0000000000000000 F F F

0.6250000000000000 0.7500000000000000 0.0000000000000000 F F F

0.8750000000000000 0.7500000000000000 0.0000000000000000 F F F

## Bidentate (2×2) 1×1

4.15597938929980

2.8284271247461801 0.0000000000000000 0.0000000000000000

0.0000000000000000 2.0000000000000000 0.0000000000000000

0.0000000000000000 0.0000000000000000 5.3033008588991004

O C H Au O C H Au

2 2 3 24 2 2 3 24

Selective dynamics

Direct

0.4717987569065555 0.5000001495677602 0.4279349786049890 T T T

0.2766492754662275 0.4999998400676169 0.4277666976948756 T T T

0.3741311413802025 0.4999998487507337 0.4531641572453660 T T T

0.3747696327320535 0.4999996542546252 0.5220075576732621 T T T

0.2879414216306114 0.4999996714170833 0.5399843294702237 T T T

0.4210867745443884 0.3935083220713183 0.5382322193961885 T T T

0.4210867848960614 0.6064909658126024 0.5382322344529891 T T T

0.0005398954742221 0.0000000426630116 0.3257469389684357 T T T

0.2489305742355438 0.0000002609111431 0.3257005334018591 T T T

0.9981875408395136 0.4999996974101957 0.3290835342714371 T T T

0.2512273333702228 0.5000012175885075 0.3289294066011484 T T T

0.1247188880640388 0.2512044692619202 0.2700804845509697 T T T

0.3747775777943748 0.2469331429312079 0.2689979358064611 T T T

0.1247187633448556 0.7487954868255441 0.2700804956285417 T T T

0.3747781174321259 0.7530679385808472 0.2689981692825993 T T T

0.0003916186271228 -0.0000001145681368 0.1964702040714152 T T T

0.2493350956498214 -0.0000001107399223 0.1964645683034584 T T T

-0.0000364970632029 0.4999996226992546 0.2023415730407370 T T T

0.2497589369310108 0.4999997842708880 0.2022633553801013 T T T

0.1249418905987787 0.2533104268470194 0.1337924418411219 T T T

0.3749603248999809 0.2533772422688890 0.1340017300269058 T T T

0.1249420179732247 0.7466890286304970 0.1337924978362985 T T T

0.3749603685980085 0.7466225793485783 0.1340019059148734 T T T

0.0000000000000000 0.0000000000000000 0.0666666666666700 F F F

0.2500000000000000 0.0000000000000000 0.0666666666666700 F F F

0.0000000000000000 0.5000000000000000 0.0666666666666700 F F F

0.2500000000000000 0.5000000000000000 0.0666666666666700 F F F

0.1250000000000000 0.2500000000000000 0.0000000000000000 F F F

0.3750000000000000 0.2500000000000000 0.0000000000000000 F F F

0.1250000000000000 0.7500000000000000 0.0000000000000000 F F F

0.3750000000000000 0.7500000000000000 0.0000000000000000 F F F

0.9718620697308664 0.4999994636833224 0.4279030584565007 T T T

0.7767138300265025 0.5000006777354489 0.4277993210652364 T T T

0.8742328070918763 0.4999999095022050 0.4531634371116770 T T T

0.8749837359520114 0.4999996438669528 0.5220039510821129 T T T

0.7881746956942590 0.4999996618374573 0.5400149085160374 T T T

0.9213461237099034 0.3935202092725182 0.5382169426623654 T T T

0.9213460736754003 0.6064791326891097 0.5382169707055989 T T T

0.5004754120425301 0.0000004951274981 0.3257707397247585 T T T

0.7489846009548276 0.0000001100830161 0.3257361640278962 T T T

0.4981667984976697 0.5000015622883210 0.3291248971778094 T T T

0.7512437154386115 0.5000022881738077 0.3289700164756347 T T T

0.6247251592224963 0.2512219336005012 0.2701424300532265 T T T

0.8747543093989191 0.2469307038347116 0.2690048576035061 T T T

0.6247247935420330 0.7487799500255560 0.2701414506779367 T T T

0.8747550478221896 0.7530699631184202 0.2690042059834944 T T T

0.5004192012699470 0.0000006029964084 0.1965133786495122 T T T

0.7493000963974644 0.0000006016722079 0.1965090884821606 T T T

0.4999884655339757 0.5000001280727093 0.2023679348781288 T T T

0.7497335799029905 0.5000001385088276 0.2022906692594563 T T T

0.6249391693149707 0.2533479892636786 0.1338044067910721 T T T

0.8749488974402364 0.2533720588820262 0.1339969038283859 T T T

0.6249389921990678 0.7466524897301431 0.1338042391018069 T T T

0.8749487767051373 0.7466278822623960 0.1339967973385413 T T T

0.5000000000000000 0.0000000000000000 0.0666666666666700 F F F

0.7500000000000000 0.0000000000000000 0.0666666666666700 F F F

0.5000000000000000 0.5000000000000000 0.0666666666666700 F F F

0.7500000000000000 0.5000000000000000 0.0666666666666700 F F F

0.6250000000000000 0.2500000000000000 0.0000000000000000 F F F

0.8750000000000000 0.2500000000000000 0.0000000000000000 F F F

0.6250000000000000 0.7500000000000000 0.0000000000000000 F F F

0.8750000000000000 0.7500000000000000 0.0000000000000000 F F F

## Bidentate c(2×2) 1×1

4.15597938929980

2.8284271247461801 0.0000000000000000 0.0000000000000000

0.0000000000000000 2.0000000000000000 0.0000000000000000

0.0000000000000000 0.0000000000000000 5.3033008588991004

O C H O C H Au O C H O C H Au

2 2 3 2 2 3 24 2 2 3 2 2 3 24

Selective dynamics

Direct

0.2217321836670089 -0.0000000017738317 0.4264212810847517 T T T

0.0265352909890464 -0.0000000007552527 0.4264014270852363 T T T

0.1241084429799623 0.0000000006086807 0.4516768888315608 T T T

0.1250421078017662 -0.0000000017157516 0.5205052775531422 T T T

0.0382718474688523 0.0000000003806351 0.5386038519929487 T T T

0.1713168517565086 0.8933623174083094 0.5366267072461159 T T T

0.1713168544026100 0.1066376835440185 0.5366267070871676 T T T

0.4717442320110285 0.4999999912079081 0.4264220757179242 T T T

0.2765403615832297 0.5000000008702188 0.4264023356133055 T T T

0.3741179393997029 0.5000000003768409 0.4516784797687010 T T T

0.3750529118416180 0.5000000077032757 0.5205122226044767 T T T

0.2882845235293062 0.5000000053886756 0.5386061978749833 T T T

0.4213253048203897 0.3933662366327204 0.5366287281052549 T T T

0.4213253121965274 0.6066337676462642 0.5366287295699366 T T T

0.0013570357303107 0.0000000030751059 0.3276412050801158 T T T

0.2476678554996093 -0.0000000007595028 0.3277357340655601 T T T

0.9976687009003760 0.4999999969682010 0.3277373250654392 T T T

0.2513604368303052 0.5000000018894928 0.3276427644295250 T T T

0.1244954281377596 0.2537073329733393 0.2680097325275290 T T T

0.3744949989596300 0.2462956155066328 0.2680077210206704 T T T

0.1244954242912302 0.7462926610588561 0.2680097425166693 T T T

0.3744949982384591 0.7537043844015472 0.2680077129475370 T T T

0.0000294607960468 0.0000000133938941 0.2004968288979082 T T T

0.2491850197821184 0.0000000078239775 0.2005486057798234 T T T

0.9991840749372448 0.4999999881555322 0.2005486401258239 T T T

0.2500319946480282 0.4999999893999479 0.2004944312001889 T T T

0.1247321757342163 0.2498367381773389 0.1332389147527098 T T T

0.3747348038551236 0.2501645480430494 0.1332378471675571 T T T

0.1247321674584083 0.7501632580934302 0.1332389154198303 T T T

0.3747348053561279 0.7498354536717993 0.1332378493452320 T T T

0.0000000000000000 0.0000000000000000 0.0666666666666700 F F F

0.2500000000000000 0.0000000000000000 0.0666666666666700 F F F

0.0000000000000000 0.5000000000000000 0.0666666666666700 F F F

0.2500000000000000 0.5000000000000000 0.0666666666666700 F F F

0.1250000000000000 0.2500000000000000 0.0000000000000000 F F F

0.3750000000000000 0.2500000000000000 0.0000000000000000 F F F

0.1250000000000000 0.7500000000000000 0.0000000000000000 F F F

0.3750000000000000 0.7500000000000000 0.0000000000000000 F F F

0.7217338769210356 -0.0000000054887742 0.4264215838345519 T T T

0.5265368984070692 0.0000000107466956 0.4264009004125510 T T T

0.6241099162866455 0.0000000015942245 0.4516768714239466 T T T

0.6250431869942331 0.0000000050711095 0.5205049790224547 T T T

0.5382727741146305 0.0000000006015547 0.5386030606759137 T T T

0.6713177912217363 0.8933623439299855 0.5366263412804283 T T T

0.6713177915024896 0.1066376596487678 0.5366263410440788 T T T

0.9717430853092122 0.5000000022290723 0.4264217075111210 T T T

0.7765386067007849 0.4999999940228614 0.4264024803953221 T T T

0.8741162633869840 0.4999999979999474 0.4516783072739395 T T T

0.8750514274865497 0.5000000002685007 0.5205118894335646 T T T

0.7882828569990538 0.5000000022151555 0.5386055417626022 T T T

0.9213238122153938 0.3933661564920226 0.5366282136351098 T T T

0.9213238121494837 0.6066338456528911 0.5366282128922792 T T T

0.5013563521732339 0.0000000075958576 0.3276412988571961 T T T

0.7476666659349079 -0.0000000026467591 0.3277362440932498 T T T

0.4976687327668142 0.4999999960502723 0.3277374351150336 T T T

0.7513581181810308 0.4999999901950172 0.3276431651206906 T T T

0.6244951276768472 0.2537062461546122 0.2680094307744583 T T T

0.8744936965594048 0.2462946965952685 0.2680097017889217 T T T

0.6244951283939537 0.7462937534623504 0.2680094375423702 T T T

0.8744936955706393 0.7537053081650469 0.2680096995573966 T T T

0.5000310204293111 0.0000000147787164 0.2004962516295166 T T T

0.7491830494592392 0.0000000122215168 0.2005487385429807 T T T

0.4991858127846512 0.4999999879703138 0.2005484000750301 T T T

0.7500315750763424 0.4999999866298910 0.2004952314777077 T T T

0.6247329070606860 0.2498373370114033 0.1332381453966818 T T T

0.8747322883714075 0.2501639276663704 0.1332383795073921 T T T

0.6247329040416507 0.7501626589052484 0.1332381446249015 T T T

0.8747322926307691 0.7498360716255087 0.1332383845530158 T T T

0.5000000000000000 0.0000000000000000 0.0666666666666700 F F F

0.7500000000000000 0.0000000000000000 0.0666666666666700 F F F

0.5000000000000000 0.5000000000000000 0.0666666666666700 F F F

0.7500000000000000 0.5000000000000000 0.0666666666666700 F F F

0.6250000000000000 0.2500000000000000 0.0000000000000000 F F F

0.8750000000000000 0.2500000000000000 0.0000000000000000 F F F

0.6250000000000000 0.7500000000000000 0.0000000000000000 F F F

0.8750000000000000 0.7500000000000000 0.0000000000000000 F F F

# PBE-TS relaxed structures:

## Chelating 1×2

4.11323373738534

2.8284271247461801 0.0000000000000000 0.0000000000000000

0.0000000000000000 2.0000000000000000 0.0000000000000000

0.0000000000000000 0.0000000000000000 5.3033008588991004

O C H Au

2 2 3 44

Selective dynamics

Direct

0.0000000095331783 0.1334235023158291 0.4239872749753396 T T T

-0.0000000017497238 0.8616522406599125 0.4239684431709566 T T T

0.0000000181215481 0.9973366792916862 0.4523570631056588 T T T

0.0000000348504477 0.9985935343480419 0.5215411613813982 T T T

0.0000000328482023 0.8745660907017903 0.5397885492413270 T T T

0.9239313002823698 0.0646114198817685 0.5380637697990086 T T T

0.0760687384312764 0.0646114313018789 0.5380637632921034 T T T

-0.0000003137091388 0.0003988350548356 0.3283338742362872 T T T

0.2534080350081032 0.0002031041274461 0.3234099098572241 T T T

0.1253181421299084 0.2538218900056460 0.2693082814475998 T T T

0.3758218650217086 0.2603777563937684 0.2716935145912758 T T T

0.1252964182261127 0.7467239505975674 0.2692712085320947 T T T

0.3758158009924158 0.7400237521812340 0.2716766781809175 T T T

-0.0000000545658814 0.0000847689911094 0.1990507703281063 T T T

0.2499228738622243 0.0001304361975886 0.1956576973574213 T T T

-0.0000000706060276 0.5001668289004183 0.2077091365640658 T T T

0.2491437417416955 0.5001629459129635 0.2079145723750334 T T T

0.1243635397868152 0.2557856744376286 0.1347636069727534 T T T

0.3745271329801833 0.2568106982095008 0.1355643708018020 T T T

0.1243788881355132 0.7442891238408430 0.1347388595739121 T T T

0.3745281372798034 0.7433468167878162 0.1355469813184765 T T T

0.0000000000000000 0.0000000000000000 0.0666666666666700 F F F

0.2500000000000000 0.0000000000000000 0.0666666666666700 F F F

0.0000000000000000 0.5000000000000000 0.0666666666666700 F F F

0.2500000000000000 0.5000000000000000 0.0666666666666700 F F F

0.1250000000000000 0.2500000000000000 0.0000000000000000 F F F

0.3750000000000000 0.2500000000000000 0.0000000000000000 F F F

0.1250000000000000 0.7500000000000000 0.0000000000000000 F F F

0.3750000000000000 0.7500000000000000 0.0000000000000000 F F F

0.4999997898768444 0.0002117228301388 0.3238289664699768 T T T

0.7465911154474686 0.0002025940012378 0.3234107756386755 T T T

0.6241784339379369 0.2603775385010197 0.2716935904033041 T T T

0.8746814136588522 0.2538210640078200 0.2693084020934619 T T T

0.6241842991461559 0.7400236335048687 0.2716767094582024 T T T

0.8747033169018360 0.7467244632133925 0.2692712923740522 T T T

0.5000000634727181 0.0001239769700289 0.1954916013737388 T T T

0.7500776804616720 0.0001305501540437 0.1956580977699843 T T T

0.5000000886227938 0.5001663564011939 0.2067516642101522 T T T

0.7508563991867636 0.5001629041779637 0.2079145507704512 T T T

0.6254730112551988 0.2568105801079804 0.1355643681444330 T T T

0.8756364470576833 0.2557856187220214 0.1347635977221969 T T T

0.6254720069727835 0.7433469550370284 0.1355469777155428 T T T

0.8756210898849816 0.7442891924008623 0.1347388527640348 T T T

0.5000000000000000 0.0000000000000000 0.0666666666666700 F F F

0.7500000000000000 0.0000000000000000 0.0666666666666700 F F F

0.5000000000000000 0.5000000000000000 0.0666666666666700 F F F

0.7500000000000000 0.5000000000000000 0.0666666666666700 F F F

0.6250000000000000 0.2500000000000000 0.0000000000000000 F F F

0.8750000000000000 0.2500000000000000 0.0000000000000000 F F F

0.6250000000000000 0.7500000000000000 0.0000000000000000 F F F

0.8750000000000000 0.7500000000000000 0.0000000000000000 F F F

## Chelating 1×1

4.11323373738534

2.8284271247461801 0.0000000000000000 0.0000000000000000

0.0000000000000000 2.0000000000000000 0.0000000000000000

0.0000000000000000 0.0000000000000000 5.3033008588991004

O C H Au

2 2 3 48

Selective dynamics

Direct

0.0000010768752365 0.1315193953546877 0.4295892586817375 T T T

0.0000017036851305 0.8597687724652627 0.4300707144812794 T T T

0.0000022550671019 0.9956414357201414 0.4582022151025046 T T T

0.0000014946428717 0.9982672926575819 0.5274002406447602 T T T

0.0000009551688914 0.8746348220929141 0.5460417551126693 T T T

0.9239315446743064 0.0645958950082658 0.5437452127546041 T T T

0.0760700535465555 0.0645962795094748 0.5437464527359216 T T T

0.9999914631188239 0.0004183099486533 0.3338754913077658 T T T

0.2542492487526909 0.0002211926523413 0.3313059491962511 T T T

0.1257518214836088 0.2491704248057511 0.2718068615046458 T T T

0.3759295690012440 0.2516454245140536 0.2729172067938974 T T T

0.1256715119406531 0.7515246782473753 0.2718121621553616 T T T

0.3759130100548345 0.7488466638910428 0.2728476315039285 T T T

0.9999983052007264 0.0001192387990883 0.2031123069416857 T T T

0.2502224677667163 0.0001908001841627 0.2002088085018686 T T T

0.9999949830959428 0.5001683309910903 0.1998188134185018 T T T

0.2498175608344673 0.5001638405004765 0.1998971803136246 T T T

0.1247045109035158 0.2490925601585017 0.1354799680378257 T T T

0.3751190604829280 0.2501313798609277 0.1361847752285370 T T T

0.1247266625494346 0.7510350116030577 0.1354529469039467 T T T

0.3751248378893553 0.7500717415131760 0.1361831937054770 T T T

0.0000000000000000 0.0000000000000000 0.0666666666666700 F F F

0.2500000000000000 0.0000000000000000 0.0666666666666700 F F F

0.0000000000000000 0.5000000000000000 0.0666666666666700 F F F

0.2500000000000000 0.5000000000000000 0.0666666666666700 F F F

0.1250000000000000 0.2500000000000000 0.0000000000000000 F F F

0.3750000000000000 0.2500000000000000 0.0000000000000000 F F F

0.1250000000000000 0.7500000000000000 0.0000000000000000 F F F

0.3750000000000000 0.7500000000000000 0.0000000000000000 F F F

0.9999946318652525 0.5002960579072687 0.3309106976793699 T T T

0.2493226115430457 0.5003790249381064 0.3315931185696314 T T T

0.5000085681171371 0.5003318827706251 0.3333920731645131 T T T

0.7506772179783304 0.5003777856705748 0.3315979906767777 T T T

0.5000054598728856 0.0001642499356609 0.3329592124595593 T T T

0.7457515820884771 0.0002200861202806 0.3313120432123173 T T T

0.6240757412123633 0.2516414398928403 0.2729141886911249 T T T

0.8742443769492563 0.2491703025311785 0.2718047796298782 T T T

0.6240948096531610 0.7488469099011872 0.2728435886894288 T T T

0.8743234173936152 0.7515217857558442 0.2718116207485419 T T T

0.5000042316051161 0.0002201163288865 0.2002782299203731 T T T

0.7497787519584475 0.0001904886666088 0.2002091839898539 T T T

0.5000015301092129 0.5001430510749365 0.2002647820717496 T T T

0.7501805387541067 0.5001653559741378 0.1998968068771860 T T T

0.6248810961005508 0.2501300920951252 0.1361812136633030 T T T

0.8752954612687978 0.2490924394287827 0.1354798382219013 T T T

0.6248747459317828 0.7500700054734983 0.1361786593967096 T T T

0.8752725283719858 0.7510411700645979 0.1354508022592702 T T T

0.5000000000000000 0.0000000000000000 0.0666666666666700 F F F

0.7500000000000000 0.0000000000000000 0.0666666666666700 F F F

0.5000000000000000 0.5000000000000000 0.0666666666666700 F F F

0.7500000000000000 0.5000000000000000 0.0666666666666700 F F F

0.6250000000000000 0.2500000000000000 0.0000000000000000 F F F

0.8750000000000000 0.2500000000000000 0.0000000000000000 F F F

0.6250000000000000 0.7500000000000000 0.0000000000000000 F F F

0.8750000000000000 0.7500000000000000 0.0000000000000000 F F F

## Bidentate top 1×2

4.11323373738534

2.8284271247461801 0.0000000000000000 0.0000000000000000

0.0000000000000000 2.0000000000000000 0.0000000000000000

0.0000000000000000 0.0000000000000000 5.3033008588991004

O C H Au

2 2 3 44

Selective dynamics

Direct

0.5239456566609619 0.9999310526261476 0.4250738321611928 T T T

0.7211918367121709 0.9999575250793323 0.4255959909717283 T T T

0.6223741606141395 0.9999432122944421 0.4509525925565905 T T T

0.6205796091304203 0.9998998743157441 0.5204268979767765 T T T

0.5736300105156349 0.1075846724766161 0.5366191203841398 T T T

0.5733836435250769 0.8924096962937489 0.5366147692675968 T T T

0.7079127864765631 0.9997098207878146 0.5391039025891156 T T T

0.0006651165232979 0.9999993897354563 0.3228042763813961 T T T

0.2465744787139140 0.9999954872122206 0.3226998916501637 T T T

0.1236559534387179 0.2616208070831689 0.2719859124965468 T T T

0.3728484894448028 0.2571798420927666 0.2699452798660608 T T T

0.1236563094346153 0.7383848957433133 0.2719803563213100 T T T

0.3728501756370192 0.7428118831155037 0.2699442232231900 T T T

0.9995009506347642 0.9999990141421967 0.1952535453222571 T T T

0.2490361485513234 0.9999990955471875 0.1952039947500743 T T T

0.9987400615951797 0.4999993797477003 0.2070351755973766 T T T

0.2488105338829534 0.4999963495628495 0.2069028585917777 T T T

0.1244669751968748 0.2567980968262917 0.1353786206216101 T T T

0.3752165650539185 0.2554791149983366 0.1347547471114933 T T T

0.1244688483017588 0.7431994781380369 0.1353763751680025 T T T

0.3752180790274365 0.7445195477165847 0.1347555204278634 T T T

0.0000000000000000 0.0000000000000000 0.0666666666666700 F F F

0.2500000000000000 0.0000000000000000 0.0666666666666700 F F F

0.0000000000000000 0.5000000000000000 0.0666666666666700 F F F

0.2500000000000000 0.5000000000000000 0.0666666666666700 F F F

0.1250000000000000 0.2500000000000000 0.0000000000000000 F F F

0.3750000000000000 0.2500000000000000 0.0000000000000000 F F F

0.1250000000000000 0.7500000000000000 0.0000000000000000 F F F

0.3750000000000000 0.7500000000000000 0.0000000000000000 F F F

0.4974011991551932 0.9999984051113459 0.3250777866735513 T T T

0.7497067787602348 0.9999990379465280 0.3254530606049891 T T T

0.6237414409860720 0.2577529554001123 0.2692512346368143 T T T

0.8743925326595711 0.2570304103252261 0.2700837429941854 T T T

0.6237413975619385 0.7422457708921187 0.2692487178242979 T T T

0.8743910415162968 0.7429710990323400 0.2700809510239611 T T T

0.4986246749971273 0.0000020376140042 0.1989516827431164 T T T

0.7499530159275277 0.0000031100338266 0.1991044002082951 T T T

0.4985306303108452 0.4999963185872054 0.2067307694986799 T T T

0.7492486278601780 0.4999979790661042 0.2066918798478043 T T T

0.6245705689048912 0.2537213165156056 0.1348489304639677 T T T

0.8738472846663318 0.2554528975878055 0.1348071262898596 T T T

0.6245706026516606 0.7462784566740635 0.1348489699999648 T T T

0.8738452686453053 0.7445472171533292 0.1348064943197545 T T T

0.5000000000000000 0.0000000000000000 0.0666666666666700 F F F

0.7500000000000000 0.0000000000000000 0.0666666666666700 F F F

0.5000000000000000 0.5000000000000000 0.0666666666666700 F F F

0.7500000000000000 0.5000000000000000 0.0666666666666700 F F F

0.6250000000000000 0.2500000000000000 0.0000000000000000 F F F

0.8750000000000000 0.2500000000000000 0.0000000000000000 F F F

0.6250000000000000 0.7500000000000000 0.0000000000000000 F F F

0.8750000000000000 0.7500000000000000 0.0000000000000000 F F F

## Bidentate top 1×1

4.11323373738534

2.8284271247461801 0.0000000000000000 0.0000000000000000

0.0000000000000000 2.0000000000000000 0.0000000000000000

0.0000000000000000 0.0000000000000000 5.3033008588991004

O C H Au

2 2 3 48

Selective dynamics

Direct

0.3922941321169734 0.0013527050163695 0.4150738947983561 T T T

0.5673579419392550 0.9988768791395358 0.4583919905969030 T T T

0.4608268954168824 0.0000014787829289 0.4629126862353968 T T T

0.3999524259469473 0.9999500638774542 0.5242252400773570 T T T

0.3442924726869800 0.1075375518701478 0.5276518192670603 T T T

0.3441257765781185 0.8924961023822009 0.5275306285561603 T T T

0.4623506994796792 0.9996484749985032 0.5618035568324969 T T T

0.9960368863764489 0.0000082271890470 0.3330000277589642 T T T

0.2390164794460347 0.9999466007334905 0.3341581427932442 T T T

0.1232792409718706 0.2504658095661226 0.2727404261740429 T T T

0.3727689226441394 0.2480636214277273 0.2721104283755529 T T T

0.1232717233639239 0.7495310218682113 0.2727403662665162 T T T

0.3727678933234826 0.7519577470340134 0.2721572443731662 T T T

0.9994106760300929 0.0000141084743665 0.2002892297366094 T T T

0.2497353946861536 0.9999984903577565 0.2021011032670085 T T T

0.0003869511201951 0.5000126170224558 0.2001429887792646 T T T

0.2493701076637788 0.5000166930599731 0.1995001957337058 T T T

0.1250000780005008 0.2487879002692217 0.1359623119965296 T T T

0.3748910586875452 0.2483866617308542 0.1358062296352505 T T T

0.1250003348604309 0.7512226841241186 0.1359602905552034 T T T

0.3748934943583184 0.7516170183299036 0.1358133232748232 T T T

0.0000000000000000 0.0000000000000000 0.0666666666666700 F F F

0.2500000000000000 0.0000000000000000 0.0666666666666700 F F F

0.0000000000000000 0.5000000000000000 0.0666666666666700 F F F

0.2500000000000000 0.5000000000000000 0.0666666666666700 F F F

0.1250000000000000 0.2500000000000000 0.0000000000000000 F F F

0.3750000000000000 0.2500000000000000 0.0000000000000000 F F F

0.1250000000000000 0.7500000000000000 0.0000000000000000 F F F

0.3750000000000000 0.7500000000000000 0.0000000000000000 F F F

0.9993830011208130 0.5000081779252326 0.3327331141820786 T T T

0.2495432539321756 0.4999791309216522 0.3313635828006587 T T T

0.4983724007852358 0.4999986805309885 0.3300522029967374 T T T

0.7486371193781980 0.5000203860688017 0.3318294657849410 T T T

0.5060057241361327 0.0000034159160645 0.3322342251489663 T T T

0.7525343582806350 0.0000288813587043 0.3318267498691978 T T T

0.6253430330569333 0.2502549623944914 0.2714317571530847 T T T

0.8747799019153559 0.2514753369149371 0.2725793688321728 T T T

0.6253239894229613 0.7498108685146946 0.2714513870337082 T T T

0.8747765024069213 0.7485505506313976 0.2725765952522757 T T T

0.5004314619395115 0.9999954531718677 0.2026103365254644 T T T

0.7505214393863738 0.0000129001019591 0.2001778185317498 T T T

0.4997418145362651 0.5000198452506313 0.1992483316184397 T T T

0.7499228605626335 0.5000317766010502 0.1997453085485569 T T T

0.6248780238317494 0.2489393896392447 0.1353684295530218 T T T

0.8752644062874338 0.2504459808409065 0.1360269289266154 T T T

0.6248791142296254 0.7510765220233442 0.1353666426838502 T T T

0.8752681171935913 0.7495870742960251 0.1360254084944539 T T T

0.5000000000000000 0.0000000000000000 0.0666666666666700 F F F

0.7500000000000000 0.0000000000000000 0.0666666666666700 F F F

0.5000000000000000 0.5000000000000000 0.0666666666666700 F F F

0.7500000000000000 0.5000000000000000 0.0666666666666700 F F F

0.6250000000000000 0.2500000000000000 0.0000000000000000 F F F

0.8750000000000000 0.2500000000000000 0.0000000000000000 F F F

0.6250000000000000 0.7500000000000000 0.0000000000000000 F F F

0.8750000000000000 0.7500000000000000 0.0000000000000000 F F F

## Bidentate (2×2) 1×2

4.11323373738534

2.8284271247461801 0.0000000000000000 0.0000000000000000

0.0000000000000000 2.0000000000000000 0.0000000000000000

0.0000000000000000 0.0000000000000000 5.3033008588991004

O C H Au O C H Au

2 2 3 22 2 2 3 22

Selective dynamics

Direct

0.2216106124079070 0.9999858933707624 0.4245175546255764 T T T

0.5243438689956550 0.9999931148614678 0.4243885710934872 T T T

0.1229144476813001 0.9999735465864950 0.4500396379259670 T T T

0.1235358462689074 0.9999557073967340 0.5195200282305503 T T T

0.5357490441978111 0.9999798797367512 0.5376410995508820 T T T

0.1703043925469752 0.8923292900032934 0.5359090718440278 T T T

0.1702982810330647 0.1075798992699205 0.5359176244457776 T T T

0.4995021752800045 0.0000062215925958 0.3245151621759121 T T T

0.2482038275758165 0.0000071660261532 0.3247150443325695 T T T

0.1241267538683790 0.2599710962238543 0.2691907959290151 T T T

0.3740909451983220 0.2564591339590861 0.2686136488563526 T T T

0.1241257307688262 0.7400394532380508 0.2691897501683605 T T T

0.3740905820820353 0.7435558971989070 0.2686114311894324 T T T

0.4993678591140079 0.0000051418767726 0.1987540514813744 T T T

0.2496293043677394 0.0000049376010679 0.1988369899504365 T T T

0.4989808699932324 0.5000046375781592 0.2059052275504309 T T T

0.2497527372171495 0.5000055690659405 0.2059050021596691 T T T

0.1247511522219051 0.2537776117895608 0.1346855571755933 T T T

0.3747461706375116 0.2538685288937871 0.1341509826747705 T T T

0.1247507297993630 0.7462254099387503 0.1346861723249622 T T T

0.3747470997209667 0.7461367751481329 0.1341507036529535 T T T

0.0000000000000000 0.0000000000000000 0.0666666666666700 F F F

0.2500000000000000 0.0000000000000000 0.0666666666666700 F F F

0.0000000000000000 0.5000000000000000 0.0666666666666700 F F F

0.2500000000000000 0.5000000000000000 0.0666666666666700 F F F

0.1250000000000000 0.2500000000000000 0.0000000000000000 F F F

0.3750000000000000 0.2500000000000000 0.0000000000000000 F F F

0.1250000000000000 0.7500000000000000 0.0000000000000000 F F F

0.3750000000000000 0.7500000000000000 0.0000000000000000 F F F

0.7215876773734149 0.9999986356020792 0.4245173270644051 T T T

0.0243664507299214 0.9999783115077334 0.4243903671853126 T T T

0.6228900133648485 0.9999901960032532 0.4500363006346363 T T T

0.6235144232443880 0.9999846585124720 0.5195198350862963 T T T

0.0357693463619987 0.9999461337649008 0.5376400720745212 T T T

0.6702849494221439 0.8923590850400643 0.5359082419889102 T T T

0.6702813439267401 0.1076107032374962 0.5359119810564722 T T T

0.9995043567769775 0.0000029447301278 0.3245203416753047 T T T

0.7482007067098077 0.0000059088126063 0.3247166398669404 T T T

0.6241266808280691 0.2599720009203041 0.2691910456590509 T T T

0.8740903949544401 0.2564480654770414 0.2686145268309909 T T T

0.6241253430759017 0.7400344414575437 0.2691905404692236 T T T

0.8740892274477375 0.7435599873400107 0.2686143889641683 T T T

0.9993706674149657 0.0000034070729261 0.1987561794184905 T T T

0.7496224828920074 0.0000036562554905 0.1988401234373191 T T T

0.9989821280718334 0.4999999717194357 0.2059084362194566 T T T

0.7497505307500903 0.5000041162132394 0.2059049691747327 T T T

0.6247521384522087 0.2537763981582884 0.1346862170310659 T T T

0.8747483154565026 0.2538678335345004 0.1341498541073906 T T T

0.6247518872499312 0.7462273530984097 0.1346869712040322 T T T

0.8747479287383625 0.7461342774201933 0.1341497594919153 T T T

0.5000000000000000 0.0000000000000000 0.0666666666666700 F F F

0.7500000000000000 0.0000000000000000 0.0666666666666700 F F F

0.5000000000000000 0.5000000000000000 0.0666666666666700 F F F

0.7500000000000000 0.5000000000000000 0.0666666666666700 F F F

0.6250000000000000 0.2500000000000000 0.0000000000000000 F F F

0.8750000000000000 0.2500000000000000 0.0000000000000000 F F F

0.6250000000000000 0.7500000000000000 0.0000000000000000 F F F

0.8750000000000000 0.7500000000000000 0.0000000000000000 F F F

## Bidentate (2×2) 1×1

4.11323373738534

2.8284271247461801 0.0000000000000000 0.0000000000000000

0.0000000000000000 2.0000000000000000 0.0000000000000000

0.0000000000000000 0.0000000000000000 5.3033008588991004

O C H Au O C H Au

2 2 3 24 2 2 3 24

Selective dynamics

Direct

0.4730662403715382 0.5000003160687594 0.4298274331738575 T T T

0.2758006178279808 0.5000005485935262 0.4297258740319948 T T T

0.3743647483495423 0.5000005654333373 0.4552995956317381 T T T

0.3750559570957562 0.4999999855405137 0.5247848460665292 T T T

0.2873023565187257 0.5000005205462935 0.5429216859890373 T T T

0.4218319248379426 0.3923730444278686 0.5411845699588869 T T T

0.4218322387434783 0.6076281681945866 0.5411846447458656 T T T

0.0002368796946280 0.0000000818647979 0.3299650284425728 T T T

0.2493438192187161 -0.0000003455550381 0.3299450953637584 T T T

0.9992296975507414 0.5000002645884388 0.3302662554772363 T T T

0.2503230171374637 0.5000011589257957 0.3301465825777054 T T T

0.1247840173521634 0.2491521926017715 0.2706974554335408 T T T

0.3748210672472339 0.2465374535378479 0.2702816945962142 T T T

0.1247832715416960 0.7508494106927974 0.2706975078599988 T T T

0.3748213554864853 0.7534606887434386 0.2702809291975685 T T T

0.0003081982622235 -0.0000001349485975 0.1982209969062457 T T T

0.2493958632135597 -0.0000017037000717 0.1982012550237484 T T T

0.9999323451537525 0.4999986471139188 0.2024464106200452 T T T

0.2497350778775916 0.4999988696859210 0.2023992413773156 T T T

0.1249011761211482 0.2523716750387952 0.1347165247518446 T T T

0.3749018741312617 0.2521906052746059 0.1350751441280074 T T T

0.1249013273883194 0.7476251670191163 0.1347166963628125 T T T

0.3749026313290894 0.7478075271602771 0.1350750001933222 T T T

0.0000000000000000 0.0000000000000000 0.0666666666666700 F F F

0.2500000000000000 0.0000000000000000 0.0666666666666700 F F F

0.0000000000000000 0.5000000000000000 0.0666666666666700 F F F

0.2500000000000000 0.5000000000000000 0.0666666666666700 F F F

0.1250000000000000 0.2500000000000000 0.0000000000000000 F F F

0.3750000000000000 0.2500000000000000 0.0000000000000000 F F F

0.1250000000000000 0.7500000000000000 0.0000000000000000 F F F

0.3750000000000000 0.7500000000000000 0.0000000000000000 F F F

0.9731481742747428 0.5000008749301831 0.4298103770493928 T T T

0.7758820880115260 0.5000005812669047 0.4297434096948549 T T T

0.8744608484754653 0.5000005709201556 0.4552993633216179 T T T

0.8752006364075324 0.4999999512164386 0.5247843207582491 T T T

0.7874667418662681 0.4999996538849510 0.5429500232487274 T T T

0.9219959140484730 0.3923769181807600 0.5411704138092387 T T T

0.9219956853795249 0.6076230151058295 0.5411707205710712 T T T

0.5002353856469310 -0.0000013494643877 0.3299593619217840 T T T

0.7493536791070131 0.0000003432210078 0.3299419859689814 T T T

0.4992071856452706 0.5000011033609262 0.3302786279262105 T T T

0.7503321214971612 0.5000006629393826 0.3301612578788025 T T T

0.6247856725376997 0.2491648712729709 0.2707160906595800 T T T

0.8748203435013130 0.2465329709110167 0.2702792329646541 T T T

0.6247863517881698 0.7508365476687850 0.2707167047857513 T T T

0.8748236072508869 0.7534665794519166 0.2702782101920928 T T T

0.5003148881187760 -0.0000003594358273 0.1982210099452682 T T T

0.7493930160700465 0.0000003425920647 0.1982044654893533 T T T

0.4999379203633317 0.5000004654519928 0.2024508546853319 T T T

0.7497390633161169 0.5000020062776063 0.2024032706908443 T T T

0.6249029048530467 0.2523697115453093 0.1347217700226425 T T T

0.8748998997694670 0.2521964485623121 0.1350692400072321 T T T

0.6249029112413539 0.7476311601881822 0.1347214415467193 T T T

0.8748999149551847 0.7478030518074631 0.1350690039811059 T T T

0.5000000000000000 0.0000000000000000 0.0666666666666700 F F F

0.7500000000000000 0.0000000000000000 0.0666666666666700 F F F

0.5000000000000000 0.5000000000000000 0.0666666666666700 F F F

0.7500000000000000 0.5000000000000000 0.0666666666666700 F F F

0.6250000000000000 0.2500000000000000 0.0000000000000000 F F F

0.8750000000000000 0.2500000000000000 0.0000000000000000 F F F

0.6250000000000000 0.7500000000000000 0.0000000000000000 F F F

0.8750000000000000 0.7500000000000000 0.0000000000000000 F F F

## Bidentate c(2×2) 1×1

4.11323373738534

2.8284271247461801 0.0000000000000000 0.0000000000000000

0.0000000000000000 2.0000000000000000 0.0000000000000000

0.0000000000000000 0.0000000000000000 5.3033008588991004

O C H O C H Au O C H O C H Au

2 2 3 2 2 3 24 2 2 3 2 2 3 24

Selective dynamics

Direct

0.2229683208614616 0.0000000473996205 0.4290861558550679 T T T

0.0256017610121218 0.0000000105110467 0.4290109377442499 T T T

0.1242392530275234 0.0000000380211264 0.4545008926730040 T T T

0.1249113977324175 0.0000000353274370 0.5239677821502722 T T T

0.0370841364319332 0.0000000244358022 0.5420349428367096 T T T

0.1715491926467693 0.8921612710577389 0.5403056278389838 T T T

0.1715492056513346 0.1078387646796378 0.5403056449854895 T T T

0.4729608853114051 0.4999999727801117 0.4290845050693527 T T T

0.2756094266999560 0.4999999680162746 0.4290138036401943 T T T

0.3742419907335237 0.4999999626825697 0.4545052635814646 T T T

0.3749143121675967 0.4999999663604310 0.5239716758279819 T T T

0.2870828726591153 0.4999999884883271 0.5420455625965576 T T T

0.4215593421515689 0.3921528547415906 0.5403136040157982 T T T

0.4215593569283725 0.6078470764613342 0.5403135899098903 T T T

0.0003203038237172 -0.0000000434690998 0.3295844985813009 T T T

0.2486353804034072 -0.0000000194511200 0.3296752495360152 T T T

0.9986494648039758 0.4999999882179584 0.3296747722329312 T T T

0.2503066552385169 0.4999999578181805 0.3295855736992545 T T T

0.1244679117587905 0.2523541160200846 0.2692246055029234 T T T

0.3744643349020317 0.2476277228211451 0.2692367921836056 T T T

0.1244679103384825 0.7476458566533737 0.2692246756064992 T T T

0.3744643206241743 0.7523722734231958 0.2692367055468642 T T T

-0.0001279601439870 0.0000000195077779 0.2010734237990366 T T T

0.2494217353791120 0.0000000124934112 0.2011082568229060 T T T

0.9994110069053924 0.5000000025582860 0.2011236462536882 T T T

0.2498618825451324 0.5000000037962580 0.2010881064261302 T T T

0.1249428329584441 0.2500316545370040 0.1342687012594580 T T T

0.3749435605441215 0.2499851010375929 0.1342787146726506 T T T

0.1249428468278480 0.7499683669866953 0.1342687388272407 T T T

0.3749435824640510 0.7500149058416490 0.1342786875474588 T T T

0.0000000000000000 0.0000000000000000 0.0666666666666700 F F F

0.2500000000000000 0.0000000000000000 0.0666666666666700 F F F

0.0000000000000000 0.5000000000000000 0.0666666666666700 F F F

0.2500000000000000 0.5000000000000000 0.0666666666666700 F F F

0.1250000000000000 0.2500000000000000 0.0000000000000000 F F F

0.3750000000000000 0.2500000000000000 0.0000000000000000 F F F

0.1250000000000000 0.7500000000000000 0.0000000000000000 F F F

0.3750000000000000 0.7500000000000000 0.0000000000000000 F F F

0.7229725257837192 0.0000000143182648 0.4290843620231857 T T T

0.5256072634990813 -0.0000000435980749 0.4290103281177421 T T T

0.6242445966527338 0.0000000054080436 0.4545001523503402 T T T

0.6249144711411926 0.0000000560289406 0.5239675186674420 T T T

0.5370873689297839 0.0000000923300739 0.5420353974034477 T T T

0.6715517246222347 0.8921625649941671 0.5403071525142610 T T T

0.6715517589103673 0.1078375337252619 0.5403071325388568 T T T

0.9729607354063226 0.4999999420591823 0.4290870182015966 T T T

0.7756096934894980 0.5000000306285650 0.4290110719025497 T T T

0.8742396620390095 0.4999999863946907 0.4545052319977971 T T T

0.8749147579455070 0.4999999965792516 0.5239715960992871 T T T

0.7870837934923154 0.5000000184196757 0.5420450099484876 T T T

0.9215597444447287 0.3921522253980261 0.5403141299252976 T T T

0.9215597541430656 0.6078477714699669 0.5403141204633914 T T T

0.5003180681179857 -0.0000000358230894 0.3295836953985398 T T T

0.7486397295828907 -0.0000000451078674 0.3296727910642302 T T T

0.4986392960037173 0.5000000241459904 0.3296708282805746 T T T

0.7503145956583518 0.5000000130062484 0.3295817354280562 T T T

0.6244668098947350 0.2523558275066717 0.2692229935469364 T T T

0.8744714162309435 0.2476279317105815 0.2692353600965390 T T T

0.6244668058048617 0.7476441615614003 0.2692230374919148 T T T

0.8744714282159238 0.7523720507336873 0.2692352587567527 T T T

0.4998641731521806 0.0000000194558321 0.2010735103106882 T T T

0.7494211550503463 0.0000000052871537 0.2011085573748013 T T T

0.4994079277599844 0.4999999801525176 0.2011251586154084 T T T

0.7498690138402087 0.4999999956002055 0.2010887632698424 T T T

0.6249425583248213 0.2500335138149699 0.1342748732241235 T T T

0.8749473791426751 0.2499818303077051 0.1342783416538498 T T T

0.6249425506254442 0.7499664779856619 0.1342748942757171 T T T

0.8749473728107635 0.7500181825980935 0.1342782983141046 T T T

0.5000000000000000 0.0000000000000000 0.0666666666666700 F F F

0.7500000000000000 0.0000000000000000 0.0666666666666700 F F F

0.5000000000000000 0.5000000000000000 0.0666666666666700 F F F

0.7500000000000000 0.5000000000000000 0.0666666666666700 F F F

0.6250000000000000 0.2500000000000000 0.0000000000000000 F F F

0.8750000000000000 0.2500000000000000 0.0000000000000000 F F F

0.6250000000000000 0.7500000000000000 0.0000000000000000 F F F

0.8750000000000000 0.7500000000000000 0.0000000000000000 F F F
